# Genome sequences of six lactic acid bacteria from artisanal cheese reveal biosynthetic gene clusters encoding antimicrobial compounds

**DOI:** 10.1128/mra.00709-24

**Published:** 2024-09-16

**Authors:** Gabriella J. Gephart, Ahmed G. Abdelhamid, Ahmed E. Yousef

**Affiliations:** 1 Department of Food Science and Technology, The Ohio State University, Columbus, Ohio, USA; 2 Botany and Microbiology Department, Faculty of Science, Benha University, Benha, Egypt; 3 Department of Microbiology, The Ohio State University, Columbus, Ohio, USA; University of Maryland School of Medicine, Baltimore, Maryland, USA

**Keywords:** lactic acid bacteria, bacteriocins, *Lactococcus*

## Abstract

Lactic acid bacteria are valuable in the production of fermented foods and as sources of antimicrobial peptides (e.g., bacteriocins). The genomes of six lactic acid bacteria, isolated from artisanal cheeses, having biosynthetic gene clusters encoding antimicrobial compounds are reported. The six strains belong to the genera *Lacticaseibacillus*, *Lactococcus*, *Leuconostoc*, and *Enterococcus*.

## ANNOUNCEMENT

Lactic acid bacteria (LAB) are widely used as starter cultures in dairy fermentations. Additionally, LAB bacteriocins are of particular interest in food preservation (1). The presence of LAB or their metabolites in fermented foods is favorably perceived by the public. Nisin, which is produced by *Lactococcus lactis*, is the only approved bacteriocin for use in the food industry (2). The emergence of resistance to nisin and its limited efficacy against Gram-negative bacteria necessitate the discovery of new bacteriocins for effective food preservation (3). Bacteriocins often inhibit bacteria closely related to the producers (4). Genome mining for antimicrobial biosynthetic gene clusters, in conjunction with antimicrobial production screening, can guide the isolation of bacteriocins that have potential uses in the food industry.

Artisanal cheeses were purchased from farmer’s markets in Columbus, Ohio, USA during the autumn of 2022. Cheese samples were homogenized, serially diluted, and spread-plated on de Man, Rogosa, and Sharpe (MRS) agar (Oxoid, Hampshire, England), and the inoculated plates were incubated at 30°C for 48 h. Colonies with unique morphologies were streaked for isolation on MRS agar. The isolates were screened for antimicrobial activity using a grid filter agar overlay method against *Listeria innocua* ATCC 33090 and *Escherichia coli* K-12 as foodborne pathogen surrogates and indicator bacteria (5). Isolates capable of inhibiting indicator bacteria were subjected to whole genome sequencing as follows: the selected isolates were cultured in MRS broth for 24 h at 30°C with shaking at 175 rpm, and the genomic DNA (gDNA) was extracted from 1.5 mL culture using a gDNA extraction kit (Qiagen QIAamp DNA Mini Kit, Qiagen, Germantown, MD) as described by the manufacturer. The purity and concentration of the gDNA were measured using a spectrophotometer (NanoVue Plus, Biochrom USA, Holliston, MA). DNA libraries were prepared using a library preparation kit (Nextera DNA Flex; Illumina, Madison, WI), indexed with a library indexing kit (Nextera DNA CD Index; Illumina), and quantified using a fluorimeter (Qubit 4; Invitrogen, Waltham, MA). The Illumina MiSeq platform (Food Microbiology Laboratory, The Ohio State University, Columbus, OH) was used to sequence the genomes, producing paired-end raw reads (2 × 150 bp). FastQC software v0.12.1 (6) was used to assess the quality of the raw reads, and the adaptors were trimmed using the “Generate FASTQ analysis module” (Illumina). The reads were used for *de novo* genome assembly using the SPAdes v3.15.3 software (7). The coverage and raw reads of the assembled genomes ranged from 38.7 × to 132.3× and 916,982 to 2,118,388, respectively (Table 1). The assembled genomes were annotated using NCBI Prokaryotic Genome Annotation Pipeline v6.7 (8). Detailed genomic characteristics for each strain are listed in Table 1. Default parameters were used for all software unless otherwise specified.

**TABLE 1:** Genomic data of the lactic acid bacteria (LAB) strains included in this study

LAB strain	Strain information									
	GenBank accession number	BioSample accession number	Raw reads accession number	FastQC Phred score	Raw reads	Coverage	Contigs (#)	N50	GC %	Total number of genes
*Lb. paracasei* OSY-31	JBDPLR000000000	SAMN41462736	SRR29540064	37	916,982	38.7×	42	357,689	46.2	3,014
*L. lactis* OSY-41	JBDPLQ000000000	SAMN41462737	SRR29540063	37	2,118,388	110.6×	115	98,282	35	2,706
*Le. mesenteroides* OSY-54	JBDPLP000000000	SAMN41462738	SRR29540062	38	2,111,676	132.3×	42	252,855	37.7	2,319
*L. lactis* OSY-92	JBDPLO000000000	SAMN41462739	SRR29540061	37	1,330,992	74.0×	135	81,303	35.1	2,701
*E. faecium* OSY-102	JBDPLN000000000	SAMN41462740	SRR29540060	37	1,087,070	59.1×	109	63,257	38	2,724
*Le. falkenbergense* OSY-112	JBDPLM000000000	SAMN41462741	SRR29540059	38	1,353,054	101.7×	109	43,259	39.1	2,020

The genomes of six LAB isolates were mined for biosynthetic gene clusters (BGCs) associated with antimicrobial production using the software antiSMASH 7.0 (9) and BAGEL4 (10). The genomes were found to contain multiple BGCs encoding ribosomally synthesized and posttranslationally modified peptides, polyketides, and class II bacteriocins. The isolates or their purified bacteriocins may have uses in the future for food preservation.

## Data Availability

The accession numbers for the genomes of the six strains are shown in Table 1. All genomes are available under BioProject accession number PRJNA1113687.
